# Risk identification of coal and gas outburst based on improved CUOWGA weighting TOPSIS model

**DOI:** 10.1038/s41598-025-00266-1

**Published:** 2025-05-12

**Authors:** Lei Wang, Baoshan Jia, Guorui Su

**Affiliations:** 1 College of Safety Science and Engineering, Liaoning Technical University, Fuxin Liaoning, 123000 China; 2 Key Laboratory of Mine Thermodynamic Disasters Prevention and Control of Ministry of Education, Huludao Liaoning, 125100 China; 3 Information Research Institute of the Ministry of Emergency Management, Chaoyang Beijing, 100029 China

**Keywords:** Coal and gas outburst, Risk assessment, Improved CUOWGA operator, TOPSIS model, Energy science and technology, Engineering

## Abstract

In order to accurately assess the risk level of coal and gas outbursts, this study proposes an evaluation method based on an improved CUOWGA-weighted TOPSIS model. The primary challenge faced in evaluating the risk of coal and gas outbursts is the subjectivity of the evaluation indicators, which may lead to unreliable outcomes. To address this issue, a coal and gas outburst evaluation indicator system comprising three key factors—geological conditions, coal seam gas content, and the physical properties of coal and rock—was constructed based on an extensive review of the literature. By introducing an innovative fuzzy semantic quantification operator and a normalized decision matrix, the computation process of the CUOWGA operator is optimized to minimize subjective bias and appropriately allocate weights to the evaluation indicators. By combining the optimized CUOWGA method with TOPSIS (Technique for Order Preference by Similarity to an Ideal Solution), the risk level of coal and gas outbursts was assessed. A case study conducted at Duanshi Coal Mine demonstrated that the risk level of coal and gas outbursts at this mine is classified as Level II, which is consistent with the actual conditions observed in the mining area. These results validate that the evaluation method based on the ICUOWGA-weighted TOPSIS model can effectively assess the risk level of coal and gas outbursts, thereby proving the feasibility of the approach.

## Introduction

As shallow resources become increasingly depleted, coal mining in China is progressively moving to greater depths^1^, facing the escalating dangers of coal and gas outbursts under conditions of high stress and high gas content. Coal and gas outbursts are dynamic manifestations of coal and rock, resulting from the nonlinear coupling effects of multiple factors such as crustal stress and gas^2–4^. These events are characterized by their sudden onset and significant harmfulness. Scientific assessment of the risk of coal and gas outbursts and the classification of their hazard levels are of paramount importance for the prevention and control of such accidents^5^.

Numerous scholars have conducted in-depth research on the evaluation of coal and gas outburst hazards and have achieved notable results. Shi Hongkai et al.^6^addressed the issue of disaster evolution not being reflected in existing studies by proposing a coal and gas outburst risk evaluation method based on combined weighting and grey clustering. Chen Liuyu and colleagues^7^, by analyzing factors influencing coal and gas outbursts, investigated the critical indices and grading criteria and developed an AHP-TOPSIS model for impact-type coal and gas outbursts. Yang Peijun et al.^8^utilized a nonlinear dynamic evaluation method to analyze the discontinuous changes and mutations of coal and gas outbursts, identifying the mutation membership degree of influencing factors at each level and proposed a coal and gas outburst evaluation method based on entropy weight mutation theory. Tang Meng and collaborators^9^ introduced the concept of game theory to optimize the subjective and objective weights of outburst indicators, constructing a coal and gas outburst risk discrimination model based on the TOPSIS method. Zhu J et al.^10^ applied rough set methods for dimensionality reduction of coal and gas outburst data to identify a set of key controlling factors. They then optimized a BP neural network using a genetic algorithm, constructing an adaptive optimization model based on genetic algorithm-back propagation (GA-BP), which enhanced the efficiency and accuracy of coal and gas outburst risk identification. Wang Wei et al.^11^ studied the influencing factors of coal and gas outbursts based on extension theory, and analyzed the subjective and objective weights of these factors using fuzzy analytic hierarchy process and correlation function methods, respectively, constructing an evaluation model based on grade correlation degree. Zhang Guorui et al.^12^ addressed the issue of inconsistent risk assessments under different conditions by proposing a coal and gas outburst evaluation model that integrates variable weight theory and uncertainty theory, effectively reducing the impact of parameter variations on weight values. Zhu Junqi et al.^13^ developed an improved quantum particle swarm optimization support vector machine (IQPSO-SVM) method to address the low accuracy of coal and gas outburst risk assessments with small samples and high-dimensional data, significantly improving the accuracy of deep coal mine outburst risk evaluations.

Although the aforementioned research achievements have advanced the development of coal and gas outburst risk assessment, several issues remain. Firstly, existing methods exhibit significant limitations in describing qualitative indicators, making it difficult to convert abstract qualitative information into actionable quantitative data. Secondly, the problem of weight allocation for quantitative indicators is prominent; traditional weighting methods often overly rely on expert experience, lacking objectivity and systematic rigor, which leads to instability in the assessment results. Furthermore, due to the complex causes of coal and gas outbursts—which involve factors such as geological stress, gas content, and the physical properties of coal and rock—there is often a high degree of nonlinearity and coupling among these factors, and current models have been unable to effectively capture these intricate interactions. In order to overcome these challenges, this paper proposes an innovative method for evaluating the risk of coal and gas outbursts.

To address these issues, this study introduces the"fuzzy semantic quantification operator“and the”optimized CUOWGA operator computation process."The fuzzy semantic quantification operator efficiently converts qualitative indicators into quantitative data, thereby enabling the assessment system to accurately reflect the influence of qualitative factors while considering quantitative indices. To mitigate the strong subjectivity inherent in traditional weight assignment methods, the proposed optimized CUOWGA operator computation process optimizes the weighting of various indicators, reducing the influence of human factors and ensuring a more rational and scientifically sound weight distribution. In addition, this paper integrates the Technique for Order of Preference by Similarity to Ideal Solution (TOPSIS) by combining the CUOWGA operator with the TOPSIS method, thus comprehensively accounting for the nonlinear coupling relationships among the influencing factors and thereby effectively enhancing the accuracy and reliability of the assessment model.

## Construction of the coal and gas outburst evaluation index system

Coal and gas outbursts are typical dynamic disasters resulting from the coupling of multiple factors such as crustal stress, coal seam gas, and the physical properties of coal and rock^7,14^. Drawing on relevant research findings, this study constructs an evaluation index system for coal and gas outbursts from three key aspects: Crustal stress conditions, coal seam gas conditions, and the physical properties of coal and rock.

**Fig. 1:**
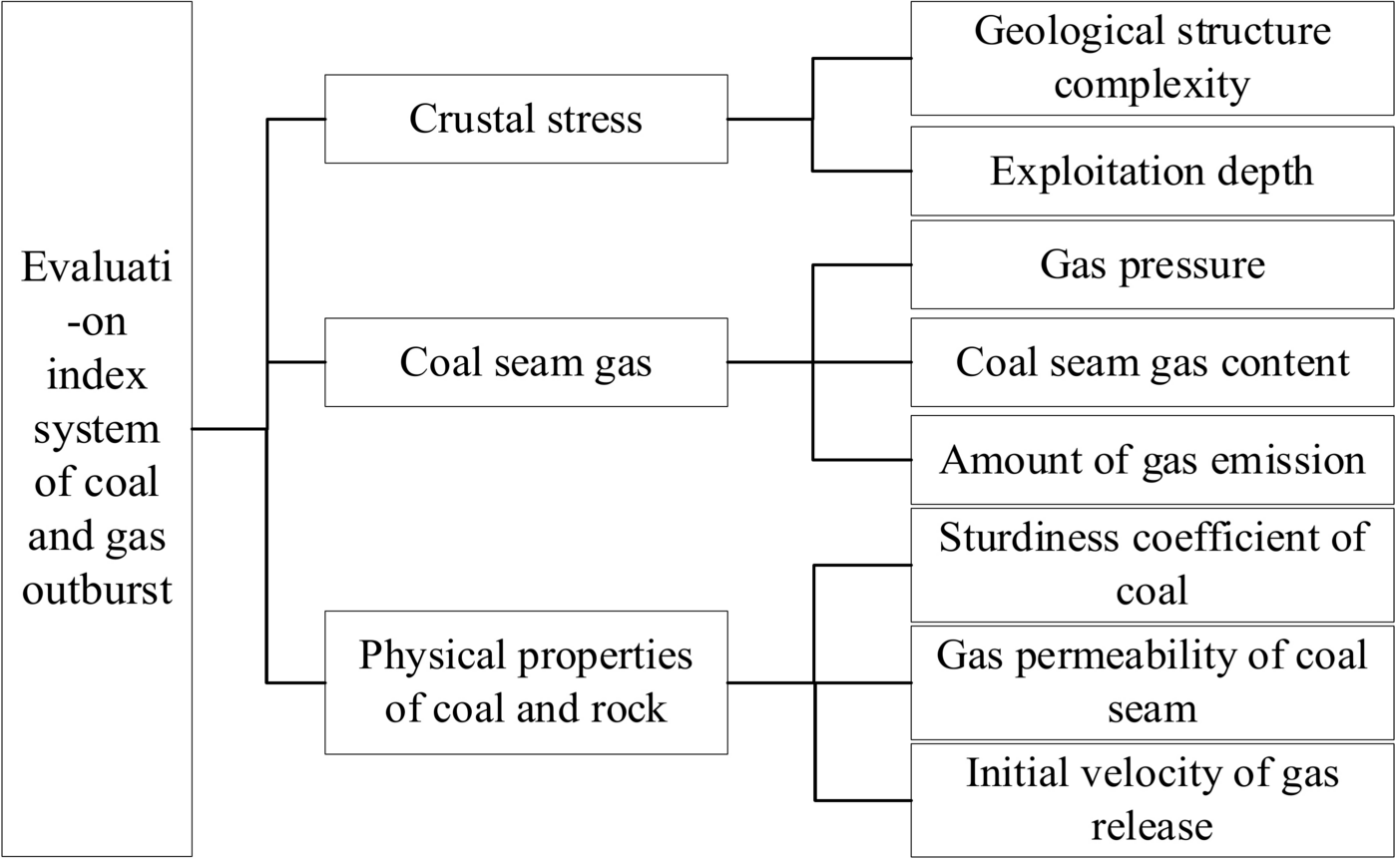
Evaluation index system of coal and gas outburst.

Crustal stress conditions are critical factors in the formation of coal and gas outbursts^15^. Higher crustal stress increases the outburst potential and the likelihood of such accidents. Key components of crustal stress conditions include the complexity of geological structures and the depth of mining. Coal seam gas conditions are the primary drivers of coal and gas outbursts^16^, with main factors including gas pressure, coal seam gas content, and the rate of gas emission. The physical properties of coal and rock determine the release carriers of coal and gas outbursts^17^, and significant factors here include the coal’s firmness coefficient, coal seam permeability, and the initial velocity of gas diffusion. Based on the above analysis, an evaluation index system for coal and gas outbursts is constructed, as illustrated in Fig. 1.

## Model construction

### Improved CUOWGA method

The traditional CUOWGA operator minimizes decision-makers’ subjective biases during decision-making, thereby improving evaluation accuracy. However, it still has the drawback of subjective condition settings^18^. This paper integrates fuzzy semantic quantification operators and a normalized decision matrix into the computation process of the CUOWGA operator to eliminate subjective influences.

The calculation process is as follows:

- (1). Determination of the weighted vector.

Assume the weighted vector is $w = (w_{1} ,w_{2} , \cdots ,w_{k} , \cdots ,w_{n} )$, then:

1 $$w_{k} = Q(\frac{k}{n}) - Q(\frac{k - 1}{n}),k \in N$$

where *Q* is the fuzzy semantic quantification operator,

2 $$Q_{(r)} = \left\{ {\begin{array}{*{20}c} {0,r < a} \\ {\frac{r - a}{{b - a}},a \le r \le b} \\ {1,r> b} \\ \end{array} } \right.$$

In the formula, according to the fuzzy semantic quantification criteria of"most,""at least half,“and”as many as possible,"the parameters of the operator *Q*_(*r*)_ are respectively:$(a,b) = (0.3,0.8)$,$(a,b) = (0,0.5)$,$(a,b) = (0.5,1)$.

- (2). Normalization of the decision matrix.

Assume the normalized matrix $R = (a_{ij} )_{m \times n}$, where $$a_{ij} = \left[ {\begin{array}{*{20}c} {a_{ij}^{L} ,a_{ij}^{U} } \\ \end{array} } \right]$$ satisfies Eqs. (3) and (4).

For cost-oriented indicators:

3 $$\left\{ {\begin{array}{*{20}c} {a_{ij}^{L} = } \\ {a_{ij}^{L} = } \\ \end{array} \frac{{\frac{{a_{ij}^{L} }}{{\sum\limits_{j = 1}^{n} {a_{ij}^{U} } }}}}{{\frac{{a_{ij}^{U} }}{{\sum\limits_{j = 1}^{n} {a_{ij}^{L} } }}}}} \right.$$

For benefit-oriented indicators:

4 $$\left\{ {\begin{array}{*{20}c} {a_{ij}^{L} = \frac{{\frac{1}{{a_{ij}^{U} }}}}{{\sum\limits_{j = 1}^{n} {(\frac{1}{{a_{ij}^{L} }})} }}} \\ {a_{ij}^{U} = \frac{{\frac{1}{{a_{ij}^{L} }}}}{{\sum\limits_{j = 1}^{n} {(\frac{1}{{a_{ij}^{U} }})} }}} \\ \end{array} } \right.$$

- (3). Construction of the possibility matrix $P = (p_{ij} )_{n \times n}$.

Among them,$p_{ij} = p(a_{i} \ge a_{j} )$, the possibility is defined as follows: Define $$a = \left[ {\begin{array}{*{20}c} {a^{L} ,a^{U} } \\ \end{array} } \right]$$,$$b = \left[ {\begin{array}{*{20}c} {b^{L} ,b^{U} } \\ \end{array} } \right]$$,$l_{a} = a^{U} - a^{L}$,$l_{b} = b^{U} - b^{L}$,then

5 $$p(a \ge b) = \frac{{\min [l_{a} + l_{b} ,\max (0,a^{U} - b^{L} )]}}{{l_{a} + l_{b} }}$$

where,$a^{L} ,a^{U}$ and $b^{L} ,b^{U}$ represent the lower and upper bounds of the possible value intervals. The values are provided based on the data distribution characteristics in coal and gas outburst scenarios, as shown in Table 1.

**Table 1:** Expert index score.

Factors	Expert 1	Expert 2	Expert 3	Expert 4	Expert 5
Crustal stress	[7,8.5]	[7.5,8.5]	[7,8.5]	[7.5,9]	[7.5,8.5]
Coal seam gas	[7.5,8.5]	[8,9]	[7.5,8.5]	[7.5,9]	[7.5,8.5]
Physical properties of coal and rock	[6,7.5]	[6.5,7.5]	[6,7.5]	[6.5,8]	[6,7.5]

- (4). Construction of the possibility complementary judgment matrix
  6 $$v_{i} = \frac{1}{n(n - 1)}\left( {\sum\limits_{j = 1}^{n} {p_{ij} + \frac{n}{2} - 1} } \right),i \in N$$
  where,$v_{i}$ represents the priority value of the alternative $i$. It is calculated as the weighted average of each row in the possibility matrix, reflecting the comprehensive possibility evaluation of coal and gas outburst risks.

- (5). Construction of the ranking vector.

All $v_{i}$ are arranged in descending order to obtain the ranking vector:

7 $$v = (v_{1} ,v_{2} , \cdots ,v_{n} )^{{\text{T}}}$$

- (6). Construction of the ICUOWGA operator.

By combining the possibility matrix and the ranking vector, the Improved Cumulative Operator Weighted Grey Approach (ICUOWGA) operator is calculated as:

8 $${\text{ICUOWGA(a}}_{{1}} {\text{,a}}_{{2}} {,} \cdots {\text{,a}}_{{\text{n}}} {) = }\prod\limits_{j = 1}^{n} {(b_{j} )^{{w_{j} }} }$$

where $w_{j}$ represents the weight vector determined, Determined through fuzzy semantic quantification methods, the specific weights are assigned based on the characteristics of coal and gas outburst scenarios (as shown in Table 2), reflecting a comprehensive evaluation of different indicators and alternatives.

**Table 2:** Index weight of coal and gas outburst.

First-grade index	Weight	Second-grade index	Weight
Crustal stress	0.592	Geological structure complexity	0.431
		Exploitation depth	0.161
Coal seam gas	0.286	Gas pressure	0.122
		Coal seam gas content	0.092
		amount of gas emission	0.072
Physical properties of coal and rock	0.122	Sturdiness coefficient of coal	0.048
		Gas permeability of coal seam	0.043
		Initial velocity of gas release	0.032

### TOPSIS model

TOPSIS^4^, a common intra-group comprehensive evaluation method, is extensively used to solve sequencing problems of optimal schemes in multi-objective decision analysis.

- (1). Establishment of the initial evaluation matrix.

Assuming that *m* evaluation objectives exist in a research topic, each consisting of *n* evaluation indexes, the following feature matrix can be constructed for the research topic:

9 $$D = \left( {\begin{array}{*{20}c} {x_{11} } & \cdots & {x_{1j} } & \cdots & {x_{1n} } \\ \vdots & \cdots & \vdots & \cdots & \vdots \\ {x_{i1} } & \cdots & {x_{ij} } & \cdots & {x_{in} } \\ \vdots & \cdots & \vdots & \cdots & \vdots \\ {x_{m1} } & \cdots & {x_{mj} } & \cdots & {x_{mn} } \\ \end{array} } \right) = \left( {\begin{array}{*{20}c} {D_{1} \left( {X_{1} } \right)} \\ \vdots \\ {D_{i} \left( {X_{i} } \right)} \\ \vdots \\ {D_{m} \left( {X_{m} } \right)} \\ \end{array} } \right)$$

where *D* is the evaluation objective; *X*_1_,*X*_2_,…,*X*_*m*_ are the evaluation schemes; and *x*_11_,*x*_*ij*_,…,*x*_*mn*_ are the evaluation indexes.

- (2). Identification of the decision matrix.

Considering various types and dimensions of indexes in the index system, the indexes are identified and nondimensionalized in accordance with different standards.

10 $$r_{ij} = \frac{{x_{ij} }}{{\sqrt {\mathop \sum \nolimits_{i = 1}^{m} x_{ij}^{2} } }}$$

where *i* = 1,2,…,*m* and *j* = 1,2,…,*n*.

- (3). Construction of the weighted decision matrix.

The weighted decision matrix is the product of the decision matrix and the corresponding weight matrix of the index system.

11 $$C = \left( {c_{ij} } \right)_{m \times n} = \left( {w_{j} r_{ij} } \right)_{m \times n} = \left( {\begin{array}{*{20}c} {w_{1} r_{11} } & \cdots & {w_{j} r_{1j} } & \cdots & {w_{n} r_{1n} } \\ \vdots & \cdots & \vdots & \cdots & \vdots \\ {w_{1} r_{i1} } & \cdots & {w_{j} r_{ij} } & \cdots & {w_{n} r_{in} } \\ \vdots & \cdots & \vdots & \cdots & \vdots \\ {w_{1} r_{m1} } & \cdots & {w_{j} r_{mj} } & \cdots & {w_{n} r_{mn} } \\ \end{array} } \right)$$

where *w*_*j*_ is the weight of index *j*, *j* = 1,2,…,n.

- (4). Calculation of the sample nearness.
- Positive and negative ideal solutions

The positive ideal solution ($A +$) and negative ideal solution ($A -$) are calculated as follows:

12 $$\left\{ {\begin{array}{*{20}c} {A^{ + } = \{ (\begin{array}{*{20}c} {maxc_{ij} } \\ i \\ \end{array} )j \in J_{1} ),(\begin{array}{*{20}c} {minc_{ij} } \\ i \\ \end{array} )j \in J_{2} ),i = 1,2,...,m = c_{1}^{ + } ,c_{2}^{ + } ,...,c_{n}^{ + } } \\ {A^{ - } = \{ (\begin{array}{*{20}c} {minc_{ij} } \\ i \\ \end{array} )j \in J_{1} ),(\begin{array}{*{20}c} {maxc_{ij} } \\ i \\ \end{array} )j \in J_{2} ),i = 1,2,...,m = c_{1}^{ - } ,c_{2}^{ - } ,...,c_{n}^{ - } } \\ \end{array} } \right.$$

where *J*_1_ represents the beneficial index set (e.g., coal mining efficiency, gas extraction efficiency, etc., which contribute positively to safety).and *J*_2_ represents the detrimental index set (e.g., gas concentration, danger index, etc., which contribute negatively to safety). $c_{ij}$ is the observed value of sample $i$ on indicator.

- Calculation of the distance between sample indexes and ideal solutions

The distance between the sample indexes and the ideal solutions can be measured by the spatial Euclidean distance.

13 $$\left\{ {\frac{{S^{ + } = \sqrt {\sum\limits_{n}^{j = 1} {(c_{ij} - c_{j}^{ + } )^{2} } } }}{{S^{ + } = \sqrt {\sum\limits_{n}^{j = 1} {(c_{ij} - c_{j}^{ - } )^{2} } } }}} \right.$$

where *S*^+^ and* S*^-^ are the Euclidean distances between the sample indexes and the positive and negative ideal solutions, *S*^+^ represents the Euclidean distance between the sample and the positive ideal solution. The larger *S*^+^ the lower the likelihood of a coal and gas outburst. *S*^-^ presents the Euclidean distance between the sample and the negative ideal solution. The smaller *S*^-^, the higher the likelihood of a coal and gas outburst.

*c*_*j*_^+^ and *c*_*j*_^-^ represent the optimal and inferior values of the indicators corresponding to the positive ideal solution (*A*^+^) and the negative ideal solution (*A*^-^), respectively. For example, *c*_*j*_^+^ could signify the lowest safe gas concentration or the maximum coal mining efficiency, while *c*_*j*_^-^ could represent the highest hazardous gas concentration or the minimum coal mining efficiency.

- Calculation of nearness
  14 $$E_{i} = \frac{{S^{ - } }}{{(S^{ + } + S^{ - } )}}$$

where $$E_{i} \in [0,1]$$.

### Risk identification

The optimal coal and gas outburst evaluation results can be obtained by combining the comprehensive weights of evaluation indexes determined by ICUOWGA and the relative nearness matrix determined by TOPSIS model.

15 $$F = \varepsilon \times E$$

where *F* is the result vector of the comprehensive evaluation model; $\varepsilon$ is the weight determined by the ICUOWGA method; and *E* is the evaluation matrix of relative nearness.

## Application validation

Using Duanshi Coal Mine as a case study, we applied the constructed coal and gas outburst evaluation index system and the ICUOWGA-TOPSIS model to evaluate coal and gas outburst risks at the mine. Duanshi Coal Mine, which is part of Qinhe Energy Group Co., Ltd., has a production capacity of 1.2 Mt/a and is currently extracting the No. 3 coal seam, identified as prone to coal and gas outbursts. The gas content of this coal seam is 12.45 m^3^/t, with a measured gas pressure of approximately 1.28 MPa. The permeability coefficient is 13.58 m^2^/MPa^2^·d, the initial gas emission velocity is 22.3 mmHg, and the coal hardness coefficient is 1.24. The type of destruction is classified as category II to III.

### Weight calculation using ICUOWGA method

Based on the coal and gas outburst evaluation index system constructed in Fig. 1, the weights are calculated using the ICUOWGA method. Five experts were engaged to score the mine’s crustal stress conditions, coal seam gas conditions, and physical properties of coal and rock. The evaluation indices are measured in multiples of 0.5, while addressing the quantification fuzziness of some indices using fuzzy numbers and interval numbers as expressions. The expert scoring data for the evaluation indices are presented in Table 1.

The following describes the process for calculating the weights of coal and gas outburst evaluation based on the ICUOWGA operator:

1. Introduce a fuzzy semantic quantification method to quantify the evaluation indices. Considering the importance of the evaluation indices, the fuzzy semantic operator is set to"as much as possible", i.e., (*l*, *h*) = (0.5,1)^19^. According to Eqs. (1) and (2), the cost-type and benefit-type indices are calculated respectively, resulting in the evaluation index weight matrix w = [0,1/3,2/3].
2. Standardization of evaluation indices. Following Eqs. (3) and (4), the expert data from Table 1 is standardized, resulting in:
  $$R = \left[ {\begin{array}{*{20}c} {\left[ {0.192,0.198} \right]} & {\left[ {0.205,0.198} \right]} & {\begin{array}{*{20}c} {\left[ {0.192,0.198} \right]} & {\left[ {0.205,0.210} \right]} & {\left[ {0.205,0.198} \right]} \\ \end{array} } \\ {\left[ {0.198,0.195} \right]} & {\left[ {0.211,0.207} \right]} & {\begin{array}{*{20}c} {\left[ {0.197,0.195} \right]} & {\left[ {0.197,0.207} \right]} & {\left[ {0.197,0.195} \right]} \\ \end{array} } \\ {\left[ {0.164,0.205} \right]} & {\left[ {0.178,0.205} \right]} & {\begin{array}{*{20}c} {\left[ {0.164,0.205} \right]} & {\left[ {0.178,0.211} \right]} & {\left[ {0.164,0.205} \right]} \\ \end{array} } \\ \end{array} } \right]$$
3. Calculation of the plausibility matrix.

By comparing the standardized matrix data according to Eqs. (5) and (6), the plausibility matrix was obtained.

$$p^{(1)} = \left[ {\begin{array}{*{20}c} {0.500} & {0.377} & {0.386} & {\begin{array}{*{20}c} {0.377} & {0.308} \\ \end{array} } \\ {0.623} & {0.500} & {0.509} & {\begin{array}{*{20}c} {0.500} & {0.424} \\ \end{array} } \\ {0.614} & {0.491} & {0.500} & {\begin{array}{*{20}c} {0.491} & {0.415} \\ \end{array} } \\ {\begin{array}{*{20}c} {0.623} \\ {0.692} \\ \end{array} } & {\begin{array}{*{20}c} {0.500} \\ {0.576} \\ \end{array} } & {\begin{array}{*{20}c} {0.509} \\ {0.585} \\ \end{array} } & {\begin{array}{*{20}c} {\begin{array}{*{20}c} {0.500} \\ {0.586} \\ \end{array} } & {\begin{array}{*{20}c} {0.424} \\ {0.500} \\ \end{array} } \\ \end{array} } \\ \end{array} } \right]$$

$$p^{(2)} = \left[ {\begin{array}{*{20}c} {0.500} & {0.479} & {0.486} & {\begin{array}{*{20}c} {0.486} & {0.573} \\ \end{array} } \\ {0.521} & {0.500} & {0.507} & {\begin{array}{*{20}c} {0.507} & {0.594} \\ \end{array} } \\ {0.514} & {0.493} & {0.500} & {\begin{array}{*{20}c} {0.500} & {0.587} \\ \end{array} } \\ {\begin{array}{*{20}c} {0.514} \\ {0.427} \\ \end{array} } & {\begin{array}{*{20}c} {0.493} \\ {0.407} \\ \end{array} } & {\begin{array}{*{20}c} {0.500} \\ {0.413} \\ \end{array} } & {\begin{array}{*{20}c} {\begin{array}{*{20}c} {0.500} \\ {0.413} \\ \end{array} } & {\begin{array}{*{20}c} {0.587} \\ {0.500} \\ \end{array} } \\ \end{array} } \\ \end{array} } \right]$$

$$p^{(3)} = \left[ {\begin{array}{*{20}c} {0.500} & {0.610} & {0.629} & {\begin{array}{*{20}c} {0.598} & {0.480} \\ \end{array} } \\ {0.390} & {0.500} & {0.520} & {\begin{array}{*{20}c} {0.488} & {0.371} \\ \end{array} } \\ {0.371} & {0.480} & {0.500} & {\begin{array}{*{20}c} {0.468} & {0.353} \\ \end{array} } \\ {\begin{array}{*{20}c} {0.402} \\ {0.520} \\ \end{array} } & {\begin{array}{*{20}c} {0.512} \\ {0.629} \\ \end{array} } & {\begin{array}{*{20}c} {0.532} \\ {0.647} \\ \end{array} } & {\begin{array}{*{20}c} {\begin{array}{*{20}c} {0.500} \\ {0.617} \\ \end{array} } & {\begin{array}{*{20}c} {0.383} \\ {0.500} \\ \end{array} } \\ \end{array} } \\ \end{array} } \right]$$

- (4). Calculation of the ranking vector.

Based on Eq. (7), the ranking vector of the plausibility matrix p(i) was computed.

$$v_{1} = \left[ {0.168,0.203,0.200,0.203,0.221} \right]$$

$$v_{2} = \left[ {0.201,0.206,0.205,0.205,0.183} \right]$$

$$v_{3} = \left[ {0.216,0.187,0.184,0.192,0.213} \right]$$

- (5). Calculation of Evaluation Index Weights.

Based on the elements’ magnitudes in the ranking vector, the decision matrix data were restructured to obtain the data gj, ordered from largest to smallest. Following Eq. (8), the ICUOWGA for ICUOWGA(U1) was calculated as [0.611, 0.573]. For simplification, the median value was taken as the weight for U1, yielding a weight of 0.592. Similarly, the ICUOWGA values for ICUOWGA(U2) and ICUOWGA(U3) were determined to be [0.305, 0.267] and [0.143, 0.101], respectively. Consequently, the weights for the evaluation indices—ground stress conditions U1, coal seam gas conditions U2, and the physical properties of coal and rock U3—are 0.592, 0.286, and 0.122, respectively.

Using the same method, the secondary index weights were recalculated. The weights for the coal and gas outburst evaluation indices, based on the ICUOWGA operator, are presented in Table 2.

### Comprehensive evaluation indexes with ICUOWGA-TOPSIS coupled model

The above indexes were classified into four grades (Grades I-IV) according to their influences on coal and gas outburst, and the indexes were graded quantitatively with references. The values of indexes were determined to be 0.2, 0.4, 0.6, 0.8 or relevant research results according to the degrees of their influences (Table 3).

**Table 3:** Classification of factors affecting coal and gas outburst.

First-grade index	Second-grade index	Influence level of index system on coal and gas outburst			
		Grade I	Grade II	Grade III	Grade IV
Crustal stress	Geological structure complexity	0.2 ≤ x < 0.4	0.4 ≤ x < 0.6	0.6 ≤ x < 0.8	x ≥ 0.8
	exploitation depth	400 ≤ x < 600	600 ≤ x < 800	800 ≤ x < 1000	x ≥ 1000
Coal seam gas	Gas pressure	0.66 ≤ x < 0.72	0.74 ≤ x < 3	3 ≤ x < 4	x ≥ 4
	Coal seam gas content	10 ≤ x < 12	12	15	x ≥ 15
	amount of gas emission	0.2 ≤ x < 0.4	0.4 ≤ x < 0.6	0.6 ≤ x < 0.8	x ≥ 0.8
Physical properties of coal and rock	Sturdiness coefficient of coal	0.6 ≤ x < 0.8	0.4 ≤ x < 0.6	0.2 ≤ x < 0.4	x ≤ 0.2
	Gas permeability of coal seam	0.2 ≤ x < 0.4	0.4 ≤ x < 0.6	0.6 ≤ x < 0.8	x ≥ 0.8
	Initial velocity of gas release	11 ≤ x < 13	13 ≤ x < 30	30 ≤ x < 40	x ≥ 40

Based on Table 3, Eq. (9), and *D*_*h*_ the initial evaluation matrix for physical properties of coal and rock established by collected related indexes of Duanshi Coal Mine, the coal and gas outburst of Duanshi Coal Mine can be evaluated:

$$D_{h} = \left[ {\begin{array}{*{20}c} \begin{gathered} 0.8 \hfill \\ 0.6 \hfill \\ 0.4 \hfill \\ 0.2 \hfill \\ 0.6 \hfill \\ \end{gathered} & \begin{gathered} 0.8 \hfill \\ 0.6 \hfill \\ 0.4 \hfill \\ 0.2 \hfill \\ 0.4 \hfill \\ \end{gathered} & \begin{gathered} 11 \hfill \\ 13 \hfill \\ 30 \hfill \\ 30 \hfill \\ 22.3 \hfill \\ \end{gathered} \\ \end{array} } \right]$$

The three physical properties of coal and rock factors in the evaluation system can be classified into two categories, i.e., beneficial and detrimental indexes. Factor Sturdiness coefficient of coal belongs to benefit index and others belong to detrimental index. On this basis, the weighted decision matrix *C*_*h*_ was constructed by combining Eqs. (10) and (11):

$$C_{h} = \left[ {\begin{array}{*{20}c} \begin{gathered} 0.013 \hfill \\ 0.026 \hfill \\ 0.394 \hfill \\ 0.394 \hfill \\ 0 \hfill \\ \end{gathered} & \begin{gathered} 0.257 \hfill \\ 0.154 \hfill \\ 0 \hfill \\ 0 \hfill \\ 0.349 \hfill \\ \end{gathered} & \begin{gathered} 0.257 \hfill \\ 0.171 \hfill \\ 0.086 \hfill \\ 0 \hfill \\ 0.171 \hfill \\ \end{gathered} \\ \end{array} } \right]$$

Based on Eq. (12), the positive and negative ideal solutions for physical properties of coal and rock factors were calculated to be $A^{ + } = \left\{ {0.394,0,0} \right\}$, and $A^{ - } = \left\{ {0,0.349,0.257} \right\}$.From Eq. (13), the distances between the positive and negative ideal solutions and each evaluation level were $S^{ + } = \left\{ {0.66,0.54,0.33} \right\}$, and $S^{ - } = \left\{ {0.55,0.46,0.77} \right\}$. From Eq. (14), the nearness of coal and gas outburst level for physical properties of coal and rock turned out $E_{m} = \left\{ {0.207,0.213,0.562,1,0.358} \right\}$.

Likewise, the nearness of coal and gas outburst level for crustal stress was calculated to be $E_{d} = \left\{ {0.262,0.281,0.368,1,0.320} \right\}$ and coal seam gas $E_{d} = \left\{ {0.272,0.293,0.433,1,0.325} \right\}$.

### Coal and gas outburst evaluation

According to the nearness of the comprehensive evaluation indexes above, the comprehensive evaluation matrix of coal and gas outburst level can be express as:

$$E = \left[ {\begin{array}{*{20}c} \begin{gathered} 0.207 \hfill \\ 0.262 \hfill \\ 0.272 \hfill \\ \end{gathered} & \begin{gathered} 0.213 \hfill \\ 0.281 \hfill \\ 0.293 \hfill \\ \end{gathered} & {\begin{array}{*{20}c} \begin{gathered} 0.562 \hfill \\ 0.368 \hfill \\ 0.433 \hfill \\ \end{gathered} & \begin{gathered} 1 \hfill \\ 1 \hfill \\ 1 \hfill \\ \end{gathered} & \begin{gathered} 0.358 \hfill \\ 0.320 \hfill \\ 0.325 \hfill \\ \end{gathered} \\ \end{array} } \\ \end{array} } \right]$$

By combining the criterion layer weights in the index system and Eq. (15), the results of the comprehensive evaluation matrix can be obtained:

$$F = \left[ {{0}{\text{.197,0}}{.372,0}{\text{.515,1,0}}{.351}} \right]$$

The quantitative classification of coal and gas outburst is listed in Table 4.

**Table 4:** Quantification of coal and gas outburst levels.

Safety risk sector	Grade I	Grade II	Grade III	Grade IV
Quantitative standard	≤ 0.197	< 0.372	< 0.515	< 1

Based on the above analysis and Table 4, the evaluation result of coal and gas outburst of Duanshi Coal Mine was determined to be 0.351, which belongs to Grade II risk. This result is consistent with the actual situation of Duanshi Coal Mine.

Sensitivity Analysis.

To ensure the robustness and accuracy of the coal and gas outburst risk assessment model, sensitivity analysis was performed by adjusting the fuzzy semantic quantification range in the CUOWGA operator. The fuzzy semantic quantification range (l = 0.5, h = 1) was adjusted to different values: l = 0.3,0.4,0.5,0.6and h = 0.8,0.9,1. The experimental results show that while these parameter configurations have some influence on the weight allocation and normalization process, the overall evaluation results remain stable. Table 5 shows the experimental settings and results for different parameter sets:

**Table 5:** The experimental settings and results for different parameter sets.

Parameter set	Experimental settings	Experimental results	Reasoning
CUOWGA Operator (Different fuzzy range settings)	Adjusting fuzzy semantic quantification range l = 0.3,0.4,0.5,0.6 and h = 0.8,0.9,1	Weight allocation and final results changed slightly, but risk grade evaluation did not show significant changes	After adjusting the range, although specific weight values were affected, the final evaluation grade remained unchanged

## Conclusions

1. This study constructed an evaluation index system for coal and gas outbursts, focusing on ground stress, coal seam gas content, and coal-rock physical properties. By introducing fuzzy semantic quantification operators and an optimized normalized decision matrix, the computation of the CUOWGA operator was refined, leading to the ICUOWGA-based method for weight assignment. Combined with the TOPSIS method, this approach effectively evaluates the risk levels of coal and gas outbursts. A case study at Duanshi Coal Mine showed that the risk level is Grade II, consistent with field conditions.
2. Compared to traditional methods, this study improves the precision and scientific accuracy of risk assessment by incorporating multiple factors and optimized operators. Many existing methods overlook the interrelations between factors, while this study adopts a systematic, multi-factor evaluation framework, enhancing the reliability of the results.
3. The proposed method accurately quantifies the risk of coal and gas outbursts, providing effective decision-making support for mine safety management. With this approach, mine managers can take early preventive measures, reducing the likelihood of accidents.
4. This method demonstrates good general applicability and accuracy but relies on region-specific coal mine data, which may limit its application in other areas. Future research can further optimize the model and validate its applicability in different mining regions.
5. This study offers an accurate risk assessment method for coal and gas outbursts, with successful practical application. Future research could combine dynamic data and real-time monitoring systems to enhance the timeliness and early-warning capabilities of the evaluation process.

## Data Availability

All data generated or analysed during this study are included in this published article.

## References

[1] Liu, W. et al. Dynamic prediction of high-temperature points in longwall gobs under a multi-field coupling framework. *Process Saf. Environ. Prot.* **187**, 1062–1075 (2024).

[2] Chong, W. A. N. G. et al. Early warning method for coal and gas outburst prediction based on indexes of deep learning model and statistical model. *Front. Earth Sci.* **10**, 811978 (2022).

[3] Liu, T. et al. Mechanical criterion for coal and gas outburst: A perspective from multiphysics coupling. *Int. J. Coal Sci. Technol.* **8**, 1423–1435 (2021).

[4] Yang, G. et al. New insights into dynamic disaster monitoring through asynchronous deformation induced coal-gas outburst mechanism of tectonic and raw coal seams. *Energy* **295**, 131063 (2024).

[5] Gaixia, C. H. E. N. Research on quantitative evaluation of contribution of coal and gas outburst prediction indicators based on machine learning. *Coal Technol.* **43**(1), 162–165 (2024).

[6] Hongkai, S. H. I. et al. Risk assessment of coal and gas outburst based on combination weighting and grey clustering. *China Saf. Sci. J.* **33**(S1), 52–57 (2023).

[7] liuyu, C. H. E. N. et al. Prediction of coal gas outburst induced by rock-burst tendency based on AHP-TOPSlS. *China Saf. Sci. J.* **30**(4), 47–52 (2020).

[8] Peijun, Y. A. N. G. et al. Risk assessment of of coal and gas outburst based on entropy weight mutation theory. *Min. Res. Dev.* **41**(7), 38–43 (2021).

[9] Meng, T. A. N. G. et al. Study on risk assessment of coal and gas outburst based on game theory and TOPSIS. *J. Shandong Univ. Sci. Technol. (Nat. Sci.)* **40**(5), 77–86 (2021).

[10] Junqi, Z. H. U. et al. Evaluation of deep coal and gas outburst based on RS-GA-BP. *Nat Hazard.* **115**(3), 2531–2551 (2023).

[11] Wei, W. A. N. G. et al. Coal and gas outburst prediction model based on extension theory and its application. *Process Saf. Environ. Prot.* **154**(329), 337 (2021).

[12] Guorui, Z. H. A. N. G. et al. A comprehensive risk assessment method for coal and gas outburst in underground coal mines based on variable weight theory and uncertainty analysis. *Process Saf. Environ. Prot.* **167**, 97–111 (2022).

[13] Junqi, Z. H. U. et al. Risk assessment of deep coal and gas outbursts based on IQPSO-SVM. *Int. J. Environ. Res. Pub. Health* **19**(19), 12869 (2022). 36232168 10.3390/ijerph191912869 PMC9564896

[14] Jiie, C. H. E. N. G. & Yi, L. I. U. Coal mine rock burst and coal and gas outburst image perception alarm method based on depth characteristics. *Coal Sci. Technol.* **52**(3), 245–257 (2024).

[15] Yongliang, H. A. N. et al. Prediction model of coal and gas outburst based on optimized GA-ELM. *Chin. J. Undergr. Sp. Eng.* **15**(6), 1895–1902 (2019).

[16] Jupeng, T. A. N. G. et al. Experimental study on precursor characteristics of coal and gas outbursts based on acoustic emission energy analysis. *Chin. J. Rock Mech. Eng.* **40**(1), 31–42 (2021).

[17] Yuhong, W. A. N. G. et al. Prediction of coal and gas outburst based on optimized quantum gated neural networks. *Inf. Control* **49**(2), 249–256 (2020).

[18] Rong, C. H. E. N. et al. Grey clustering evaluation of building construction safety based on CUOWGA operator weighting. *Saf. Environ. Eng.* **27**(5), 98–105 (2020).

[19] Chuanlian, C. H. E. N. & Jianqin, W. A. N. G. Research on the risk assessment of high speed railway project construction based on improved CUOWGA-Shapley value. *Constr. Econ.* **38**(11), 43–46 (2017).

